# CMG-Biotools, a Free Workbench for Basic Comparative Microbial Genomics

**DOI:** 10.1371/journal.pone.0060120

**Published:** 2013-04-05

**Authors:** Tammi Vesth, Karin Lagesen, Öncel Acar, David Ussery

**Affiliations:** 1 Center for Biological Sequence Analysis, Department of Systems Biology, Technical University of Denmark, Kgs. Lyngby, Denmark; 2 Centre for Ecological and Evolutionary Synthesis, Department of Biology, University of Oslo, Oslo, Norway; 3 Department of Electrical Engineering, Technical University of Denmark, Kgs. Lyngby, Denmark; University of Florida, United States of America

## Abstract

**Background:**

Today, there are more than a hundred times as many sequenced prokaryotic genomes than were present in the year 2000. The economical sequencing of genomic DNA has facilitated a whole new approach to microbial genomics. The real power of genomics is manifested through comparative genomics that can reveal strain specific characteristics, diversity within species and many other aspects. However, comparative genomics is a field not easily entered into by scientists with few computational skills. The CMG-biotools package is designed for microbiologists with limited knowledge of computational analysis and can be used to perform a number of analyses and comparisons of genomic data.

**Results:**

The CMG-biotools system presents a stand-alone interface for comparative microbial genomics. The package is a customized operating system, based on Xubuntu 10.10, available through the open source Ubuntu project. The system can be installed on a virtual computer, allowing the user to run the system alongside any other operating system. Source codes for all programs are provided under GNU license, which makes it possible to transfer the programs to other systems if so desired. We here demonstrate the package by comparing and analyzing the diversity within the class *Negativicutes*, represented by 31 genomes including 10 genera. The analyses include 16S rRNA phylogeny, basic DNA and codon statistics, proteome comparisons using BLAST and graphical analyses of DNA structures.

**Conclusion:**

This paper shows the strength and diverse use of the CMG-biotools system. The system can be installed on a vide range of host operating systems and utilizes as much of the host computer as desired. It allows the user to compare multiple genomes, from various sources using standardized data formats and intuitive visualizations of results. The examples presented here clearly shows that users with limited computational experience can perform complicated analysis without much training.

## Introduction

The number of microbial genome sequences has exploded due to the lower cost of sequencing facilitated by advances in sequencing technology making these services easier and faster. There are now roughly a hundred times as many sequenced prokaryotic genomes available as in 2000. The National Center for Biotechnology Information (NCBI) has an online list of genome sequences, complete and in progress. In 2000, 42 sequenced genomes were available on the NCBI list, and this number had grown to 4 189 in February 2012 (www.ncbi.nlm.nih.gov/genomes/lproks.cgi). Further, recently a single study [1] has compared genome sequences from 2 348 *Mycobacterium tuberculosis* isolates, and there are many more studies in progress where thousands of bacterial genome sequences are compared. As a consequence, more experimental biologists with little to no experience with bioinformatics find themselves in possession of an enormous amount of sequencing data and in need of tools necessary for analysis.

Analyzing the sequence of a single genome can confer a wide range of knowledge [2], [3]. It is possible to use alignment tools to find a specific gene in a genome within seconds, for example to identify a genetic marker for a specific phenotype. DNA structure analyses can pinpoint chromosomal regions that lend themselves to certain genes and genomic elements. Regions that show distinct structural properties along the chromosome include clusters of genes encoding surface-proteins (usually more AT rich), possible phage insertions, regions likely to contain highly expressed genes as well as potential genomic islands [4]–[6]. Based on the annotation of a genome it is also possible to find the gene neighbors of a specific gene, thus possibly identifying functionally connected genes. The sequencing of individual genomes has facilitated a whole new approach to wet lab experiments that until recently were not possible. There is an enormous amount of information just in a single genome sequence.

However, the real power of genomics is manifested through comparative genomics. Even within a species, comparative genomics has highlighted a diversity that would not have been detected otherwise. The diversity within *Escherichia coli* was illustrated in a study from 2009, where the number of gene families, in *Escherichia coli* was estimated to be 43 000 [7]; this number is expected to become larger as more genomes are sequenced. Another example of the power of comparative genomics, this time within low diversity genomes, can be found in a study of two *Bacillus* species, *B. anthracis* and *B. cereus*. These are difficult to differentiate based on chromosomal markers [8], and the difference in pathogenicity is solely determined by the strict presence of two virulence plasmids, which both are required for anthrax. The diversity of a species can be estimated by multiple sequence comparisons across genomes calculating the pan genome (all genes found in genomes) [9]. Comparative microbial genomics (CMG) also allows for fast and inexpensive analyses, for example phylogenetic relationships between organisms. Further, it is possible to build up data from known organisms that would allow for quick classification of an isolate of an unknown organism, just from its genome sequence.

The CMG-biotools package presented here is designed for microbiologists with limited knowledge of computational analyses and comes with a basic introduction to Unix. Within this package it is possible to do phylogenetic analysis, proteome comparisons, DNA structure analysis and much more, just with a list of genomes. Most of the analyses can be performed on FASTA formatted DNA sequences from unpublished projects as well. The CMG-biotools system presents a stand-alone interface for comparative microbial genomics. The package is a installable operating system, based on Xubuntu 10.10 available through the open source Ubuntu project (www.xubuntu.org/get). This setup overcomes problems with dependencies and platform specificity allowing for all users to work in the same environment. Ubuntu is a widely used, free of charge and open source operating system with a large user community and thousands of free applications. As of 2012, Ubuntu is the second most popular Linux distribution, only surpassed by Mint [10]. It is a stand-alone operating system and can be installed directly onto a local computer or on a virtual computer using virtualization software. The CMG-biotools operating system has been tested on a free virtualization application, VirtualBox (www.virtualbox.org). This system addresses the problem of working with large amounts of data, allowing for comparative analyses of multiple genomes, thereby making use of the vast amount of sequence information that is now available in laboratories all over the world.

## Results and Discussion

**Figure 1:**
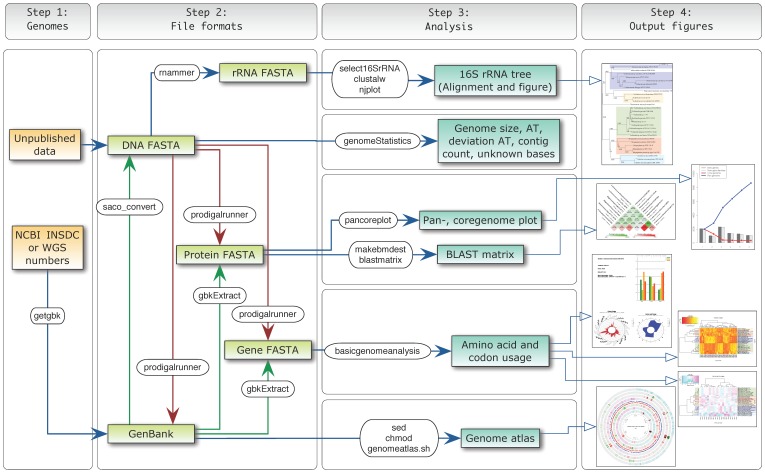
Analysis workflow. Visual representation of the data flow through each of the steps in the CMG-biotools system. The figure shows the analysis input and program name along with the analysis output type. Green arrows indicate data extraction from a GenBank file format, this data needs to be available in the file for these steps to work. Red arrows indicate local genefinding which results in gene FASTA, protein FASTA and GenBank files.

To demonstrate the capabilities of the CMG-biotools (Comparative Microbial Genomics), analyses are performed on a set of genomes from the class *Negativicutes*. The CMG-biotools operating system was installed on an 8 Gigabyte virtual computer using VirtualBox (www.virtualbox.org). Figure 1 illustrates the work and data flow of the analyses.

### Data Collection and Assessment

The first step of the analyses is to obtain genome data for a set of organisms. In the example presented here, we obtain data from the GenBank database [11] at the National Center for Biotechnology Information (NCBI, www.ncbi.nlm.nih.gov/genome/browse/) This database is part of the International Nucleotide Sequence Database Collaboration (INSDC) and contains more than 3000 bacterial genome projects. For the example, organisms of the class *Negativicutes* were identified from NCBI genomes list (www.ncbi.nlm.nih.gov/genome/browse/, “Prokaryotes”, *Negativicutes* (taxid:909932)) and GenBank INSDC numbers or whole genome sequence numbers (WGS) were obtained. The genome sequences of 6 complete (NCBI Genomes list, status: “Complete”) and 25 assembly genomes (NCBI Genomes list “Scaffolds/contigs”) were identified. NCBI GenBank INSDC numbers were used for complete genomes while WGS numbers were used for draft sequences. Using the program getgbk and the INSDC/WGS numbers, each genome was downloaded in the NCBI GenBank format (Figure 1, Step 1). A list of genome names and INSDC/WGS numbers is found in Table 1. DNA sequences were extracted from GenBank files and saved in FASTA format(*saco_conver t* [12], Figure 1, Step 2B).

**Table 1:** Genome information.

Tax	Organism	INSDC	WGS	WGS for download	Status
591001	*Acidaminococcus fermentans* DSM 20731	CP001859	–	–	Complete
568816	*Acidaminococcus intestini* RyC-MR95	CP003058	–	–	Complete
563191	*Acidaminococcus sp* D21	–	ACGB01	ACGB00000000	Scaffolds/contigs
888060	*Centipeda periodontii* DSM 2778	–	AFHQ01	AFHQ00000000	Scaffolds/contigs
592028	*Dialister invisus* DSM 15470	–	ACIM02	ACIM00000000	Scaffolds/contigs
888062	*Dialister micraerophilus* DSM 19965	–	AFBB01	AFBB00000000	Scaffolds/contigs
910314	*Dialister microaerophilus* UPII 345-E	–	AENT01	AENT00000000	Scaffolds/contigs
158847	*Megamonas hypermegale* ART12 1	FP929048	–	–	Complete
907	*Megasphaera elsdenii* DSM 20460	HE576794	–	–	Complete
699218	*Megasphaera genomosp* type 1 str 28L	–	ADGP01	ADGP00000000	Scaffolds/contigs
706434	*Megasphaera micronuciformis* F0359	–	AECS01	AECS00000000	Scaffolds/contigs
1000569	*Megasphaera sp* UPII 135-E	–	AFUG01	AFUG00000000	Scaffolds/contigs
1000568	*Megasphaera sp* UPII 199-6	–	AFIJ01	AFIJ00000000	Scaffolds/contigs
500635	*Mitsuokella multacida DSM 20544*	–	ABWK02	ABWK00000000	Scaffolds/contigs
626939	*Phascolarctobacterium succinatutens*YIT 12067	–	AEVN01	AEVN00000000	Scaffolds/contigs
749551	*Selenomonas artemidis* F0399	–	AECV01	AECV00000000	Scaffolds/contigs
638302	*Selenomonas flueggei* ATCC 43531	–	ACLA01	ACLA00000000	Scaffolds/contigs
585503	*Selenomonas noxia* ATCC 43541	–	ACKT01	ACKT00000000	Scaffolds/contigs
879310	*Selenomonas sp* oral taxon 137 str F0430	–	AENV01	AENV00000000	Scaffolds/contigs
864563	*Selenomonas sp* oral taxon 149 str 67H29BP	–	AEEJ01	AEEJ00000000	Scaffolds/contigs
546271	*Selenomonas sputigena* ATCC 35185	CP002637	ACKP02	ACKP00000000	Complete
401526	*Thermosinus carboxydivorans* Nor1	–	AAWL01	AAWL00000000	Scaffolds/contigs
866776	*Veillonella atypica* ACS-049-V-Sch6	–	AEDR01	AEDR00000000	Scaffolds/contigs
866778	*Veillonella atypica* ACS-134-V-Col7a	–	AEDS01	AEDS00000000	Scaffolds/contigs
546273	*Veillonella dispar* ATCC 17748	–	ACIK02	ACIK00000000	Scaffolds/contigs
686660	*Veillonella parvula* ATCC 17745	–	ADFU01	ADFU00000000	Scaffolds/contigs
479436	*Veillonella parvula* DSM 2008	CP001820	–	–	Complete
457416	*Veillonella sp* 3 1 44	–	ADCV01	ADCV00000000	Scaffolds/contigs
450749	*Veillonella sp* 6 1 27	–	ADCW01	ADCW00000000	Scaffolds/contigs
879309	*Veillonella sp* oral taxon 158 str F0412	–	AENU01	AENU00000000	Scaffolds/contigs
944564	*Veillonella sp* oral taxon 780 str F0422	–	AFUJ01	AFUJ00000000	Scaffolds/contigs

Table listing the genomes used in the analysis. Data was downloaded from NCBI GenBank database. Abbreviations: *Tax*: NCBI taxonomy id number, *Organism*: Name of organism, *INSDC*: NCBI GenBank Accession number, *WGS*: NCBI Whole Genome Sequence Project number, *Status*: status of sequencing project. The WGS number can be used for downloading whole genome sequencing projects by removing the last two numbers and adding 6 zeros (ACGB01 is downloaded using the number ACGB000000).

Basic statistical parameters were calculated for the 31 genomes (Figure 1, Step 3B), using whole genome DNA FASTA files as input, and the results are shown in Table 2. The AT content varied from 42 to 66% and the genome size ranged from 1.26 to 2.89 Mega bases (Mb). The percentage of unknown bases refers to letters in the DNA code that are not A, C, T or G. These bases might be the result of an assembly process or errors in sequencing. Of the 31 genomes analyzed, 8 had non-canonical base letters in the DNA sequences, ranging from 0.0001%. to 3.6%. The fraction of the largest contig will be 100% for genomes with one chromosome and therefore this measure is more useful for identification of incomplete sequences. For the non-complete genomes, the fraction made up by the largest sequence varied from 5% to 30%. It is seen the the fraction correlates with the number of contigs, if the genome sequence is in many contigs, then the largest sequence covers a small fraction of the entire genome. These findings show a large variation in the dataset, both in the context of biology (AT content and size) and data quality (number of contigs and percentage of unknown bases).

**Table 2:** Genome statistics.

Organism	bp	AT	Std. AT	Contig	Unknown	Largest	N50
*Acidaminococcus fermentans* DSM 20731	2 329 769	44,16	–	1	–	100	2 329 769
*Acidaminococcus intestini* RyC-MR95	2 487 765	49,98	–	1	–	100	2 487 765
*Acidaminococcus sp* D21	2 238 973	49,80	0,03	79	–	6,2	43 082
*Centipeda periodontii* DSM 2778	2 650 230	44,02	0,04	71	–	8,4	72 349
*Dialister invisus* DSM 15470	1 895 860	54,50	0,03	2	–	99,9	1 894 898
*Dialister micraerophilus* DSM 19965	1 256 198	64,69	0,05	32	–	17,9	90 852
*Dialister microaerophilus* UPII 345-E	1 395 825	64,35	0,07	32	–	15,4	122 970
*Megamonas hypermegale* ART12 1	2 209 938	65,89	–	1	3,602	100	2 209 938
*Megasphaera elsdenii* DSM 20460	2 474 718	47,01	–	1	0,397	100	2 474 718
*Megasphaera genomosp* type 1 str 28L	1 726 197	53,95	0,03	34	–	12,2	156 177
*Megasphaera micronuciformis* F0359	1 765 374	54,56	0,04	49	–	24,8	142 252
*Megasphaera sp* UPII 135-E	1 440 762	61,19	0,04	46	0,001	12,0	63 822
*Megasphaera sp* UPII 199-6	1 242 998	53,26	0,04	38	–	12,0	96 055
*Mitsuokella multacida* DSM 20544	2 204 718	41,89	0,04	28	–	19,5	321 943
*Phascolarctobacterium succinatutens* YIT 12067	2 122 261	52,36	0,05	118	–	5,1	43 220
*Selenomonas artemidis* F0399	2 209 623	42,75	0,06	66	–	19,7	89 528
*Selenomonas flueggei* ATCC 43531	2 157 862	44,03	0,04	33	–	12,2	125 841
*Selenomonas noxia* ATCC 43541	2 039 467	44,13	0,05	56	–	14,2	106 401
*Selenomonas sp* oral taxon 137 str F0430	2 475 066	43,27	0,05	15	–	22,1	306 540
*Selenomonas sp* oral taxon 149 str 67H29BP	2 429 414	43,20	0,05	56	–	7,8	95 526
*Selenomonas sputigena* ATCC 35185	2 568 361	42,89	–	1	–	100	2 568 361
*Thermosinus carboxydivorans* Nor1	2 889 774	48,50	0,03	49	–	12,1	108 262
*Veillonella atypica* ACS-049-V-Sch6	2 053 871	61,03	0,04	63	–	10,3	80 793
*Veillonella atypica* ACS-134-V-Col7a	2 151 913	61,02	0,04	70	–	9,8	74 331
*Veillonella dispar* ATCC 17748	2 116 567	61,14	0,06	25	–	30,4	498 249
*Veillonella parvula* ATCC 17745	2 163 473	61,43	0,04	19	–	26,9	416 853
*Veillonella parvula* DSM 2008	2 132 142	61,37	–	1	–	100	2 132 142
*Veillonella sp* 3 1 44	2 156 561	61,36	0,04	31	–	18,0	282 953
*Veillonella sp* 6 1 27	2 169 785	61,33	0,04	22	–	15,8	257 597
*Veillonella sp* oral taxon 158 str F0412	2 176 752	61,05	0,04	21	–	19,3	366 615
*Veillonella sp* oral taxon 780 str F0422	1 731 014	60,55	0,03	75	–	14,0	73 892

Basic genome statistics for genome DNA sequences. Values of zero are marked by “−”. Abbreviations: *Organism*: Name of organism. *Status*: sequencing status of published project. *bp*: total number of base pairs in all DNA. *AT*: Percent of AT in DNA. *Std. AT*: Standard deviation in AT across DNA fragments. *Contig*: number of DNA fragments corresponding to replicons or contigs. *Unknown*: percentage of unknown bases (not A, T, C or G). *Largest*: size of largest contig as a percentage of total length. *N50*: weighted median statistic such that 50% of the entire assembly is contained in contigs or scaffolds equal to or larger than this value.

### Gene Finding

The next step in the analysis is to identify coding regions in DNA sequences. Some genome projects have manually curated and high quality annotations while others have no annotations at all. Again others have been annotated using a genefinding algorithm without any additional evaluation of the findings. The CMG-biotools uses the program Prodigal [13] for genefinding and has been incorporated into a pipeline called prodigalrunner. This pipeline takes a genome DNA GenBank or FASTA file as input (Figure 1, Step 2C) The output from prodigalrunner is a preliminary GenBank file (.gbk), a general feature format file (.gff), a FASTA formatted open reading frame file (genes,.orf.fna) and a FASTA formatted protein file which contains the translations of the genes (orf.fsa). Table 3 shows the number of published genes compared to the number of genes found when using Prodigal for genefinding.

**Table 3:** Genefinding and published genes.

Genome name	GenBank	Prodigal	ID
*Acidaminococcus fermentans* DSM 20731	2 026	2 063	CP001859
*Acidaminococcus intestini* RyC-MR95	2 404	2 372	CP003058
*Acidaminococcus sp* D21	2 005	2 105	ACGB00000000
*Centipeda periodontii* DSM 2778	2 559	2 440	AFHQ00000000
*Dialister invisus* DSM 15470	1 954	1 765	ACIM00000000
*Dialister micraerophilus* DSM 19965	1 243	1 206	AFBB00000000
*Dialister microaerophilus* UPII 345-E	1 310	1 308	AENT00000000
*Megamonas hypermegale* ART12 1	2 118	2 759	FP929048
*Megasphaera elsdenii* DSM 20460	2 220	2 222	HE576794
*Megasphaera genomosp* type 1 str 28L	1 610	1 560	ADGP00000000
*Megasphaera micronuciformis* F0359	1 774	1 724	AECS00000000
*Megasphaera sp* UPII 135-E	1 310	1 291	AFUG00000000
*Megasphaera sp* UPII 199-6	1 151	1 112	AFIJ00000000
*Mitsuokella multacida* DSM 20544	2 142	1 942	ABWK00000000
*Phascolarctobacterium succinatutens* YIT 12067	2 150	2 012	AEVN00000000
*Selenomonas artemidis* F0399	2 195	2 024	AECV00000000
*Selenomonas flueggei* ATCC 43531	2 117	2 045	ACLA00000000
*Selenomonas noxia* ATCC 43541	2 020	1 955	ACKT00000000
*Selenomonas sp* oral taxon 137 str F0430	2 395	2 341	AENV00000000
*Selenomonas sp* oral taxon 149 str 67H29BP	2 407	2 313	AEEJ00000000
*Selenomonas sputigena* ATCC 35185	2 255	2 283	CP002637
*Thermosinus carboxydivorans* Nor1	2 750	2 886	AAWL00000000
*Veillonella atypica* ACS-049-V-Sch6	1 840	1 865	AEDR00000000
*Veillonella atypica* ACS-134-V-Col7a	1 903	1 923	AEDS00000000
*Veillonella dispar* ATCC 17748	1 954	1 941	ACIK00000000
*Veillonella parvula* ATCC 17745	1 929	1 945	ADFU00000000
*Veillonella parvula* DSM 2008	1 844	1 865	CP001820
*Veillonella sp* 3 1 44	0	1 922	ADCV00000000
*Veillonella sp* 6 1 27	0	1 936	ADCW00000000
*Veillonella sp* oral taxon 158 str F0412	2 000	2 029	AENU00000000
*Veillonella sp* oral taxon 780 str F0422	1 588	1 605	AFUJ00000000

Table listing genome name, number of published proteins (*GenBank*) and number of proteins found using Prodigal for genefinding (*Prodigal*). The column labeled *“ID”* refers to the INSDC or WGS id number as described in Table 1.

This genefinder found between 1 206 (*D. micraerophilus* DSM 19965) and 2 886 (*Thermosinus carboxydivorans* Nor1) proteins in the 31 genomes. Compared to the published proteins from GenBank, Prodigal finds roughly the same number of genes, except for two genomes which did not have any published annotations. The advantage of using an independent gene finder for all genome sequences in an analysis is that the difference introduced by annotators will be removed. As information on how genefinding was performed is rarely available, doing local genefinding might eliminate badly annotated projects. Whether to use published annotations is up to the individual user but for obvious reasons, genefinding will have to be done for projects with no published annotations. For the remaining analysis in this paper, proteomes predicted using prodigalrunner will be used.

### Phylogenetic Analysis

**Figure 2:**
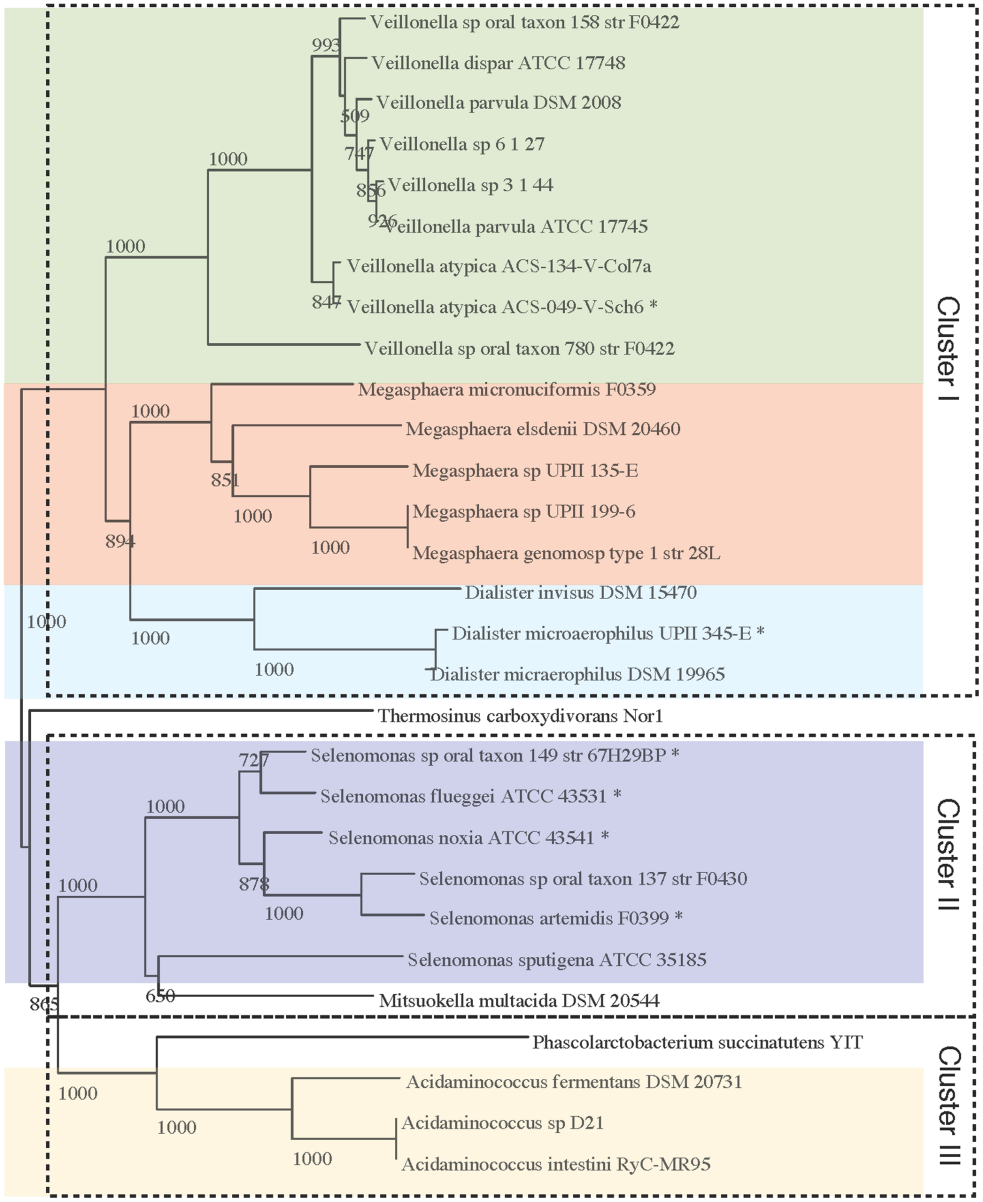
16S rRNA tree. Each genome sequence was searched for 16S rRNA patterns and candidate sequences were extracted. The best sequence from each genome was selected. For two genomes, no sequences were found, *Centipeda periodontii* DSM 2778, *Megamonas hypermegale* ART12 1. For 6 additional genomes, the located sequences were shorter than the default acceptable length. The short sequences sequences are marked with a “*”. Length criteria was changed from minimum 1 400 to 1 100 and maximum 1 800 unchanged. The distance tree was made with 1 000 bootstraps.

The chromosomal DNA sequence, as extracted from the GenBank files (FASTA format) is used as input for this analysis, as illustrated in Figure 1, Step 2A. The whole genome DNA sequence is searched for rRNA sequences using RNAmmer [14] and a sequence from each genome is extracted (select16SrRNA, Figure 1, Step 3A). The selection criteria for the extraction process defaults to the highest scoring sequence found with a length between 1 400 and 1 800 base pairs. This selection is not necessarily the most correct way of selecting a 16S rRNA sequence for phylogenetic analysis, but offers the opportunity to compare genomes based on a single sequence. The alignment program ClustalW [15] is used for multiple sequence alignment of the sequences. From the alignment, a distance tree is constructed, using 1 000 bootstraps [16] to find the best fitting distance tree (the output is a file Phylip tree format.phb). Each node of the tree is shown with a bootstrap value between 0 and 1 000, the number indicating how many times this branching is seen out of 1 000 re-samplings. The higher the number the more reliable the branching. The visualization of the tree was done using njplot [17] and is shown in Figure 2.

The results of the RNAmmer analysis yielded no rRNA sequences for two genomes (*Centipeda periodontii* DSM 2778, 72 contigs, and *Megamonas hypermegale* ART12 1, 1 replicon). Sequences from 6 genomes had lengths outside the default thresholds - length between 1 400 and 1 800 base pairs (Table 4, 16S rRNA length and score for each genome). For this analysis the thresholds were changed to include these 6 genomes (lower threshold for sequence length was changed to 1 100 base pairs). The genome of *Megamonas hypermegale* contains a large number of unknown bases (found in 99 stretches of lengths between 141 and 1780 nucleotides, calculated using countUnknowns.pl). The average length of these stretches was 804 nucleotide positions, roughly half the length for a 16S rRNA sequence. It is here hypothesized that such unknown base stretches can prevent rnammer from identifying ribosomal RNA sequences, because parts of the 16S rRNA sequence might be missing. The sequence of *Centipeda periodontii* DSM 2778 does not contain any unknown bases, but still no rRNA sequences were found in this sequence. The genome is in 72 contigs and the largest sequence is 8.5% of the total, numbers that are not extreme compared to other genomes in this analysis (Table 3). It can be hypothesized that the lack of 16S rRNA sequences in this genome might be a result of the sequence assembly. Since ribosomal RNA sequences often are repeated sequences, the assembly process might not be able to conclusively place the rRNA in the DNA, and might discard the sequences all-together.

**Table 4:** Ribosomal RNA analysis using RNAmmer.

Organism	Status	Score	Length (bp)	Total seq.
*Acidaminococcus fermentans* DSM 20731	Complete	1 910.8	1 545	6
*Acidaminococcus intestini* RyC-MR95	Complete	1 920.1	1 545	3
*Acidaminococcus sp* D21	Scaffolds/contigs	1 920.1	1 545	1
*Centipeda periodontii* DSM 2778	Scaffolds/contigs	–	–*	–
*Dialister invisus* DSM 15470	Scaffolds/contigs	1 836.1	1 557	3
*Dialister micraerophilus* DSM 19965	Scaffolds/contigs	1 878.8	1 555	1
*Dialister microaerophilus* UPII 345-E	Scaffolds/contigs	1 197.2	1 325*	1
*Megamonas hypermegale* ART12 1	Complete	–	–*	–
*Megasphaera elsdenii* DSM 20460	Complete	1 842.0	1 552	7
*Megasphaera genomosp* type 1 str 28L	Scaffolds/contigs	1 860.0	1 557	1
*Megasphaera micronuciformis* F0359	Scaffolds/contigs	1 816.0	1 550	1
*Megasphaera sp* UPII 135-E	Scaffolds/contigs	1 887.4	1 556	1
*Megasphaera sp* UPII 199-6	Scaffolds/contigs	1 868.7	1 556	1
*Mitsuokella multacida* DSM 20544	Scaffolds/contigs	1 915.8	1 549	2
*Phascolarctobacterium succinatutens* YIT 12067	Scaffolds/contigs	1 907.9	1 646	1
*Selenomonas artemidis* F0399	Scaffolds/contigs	6.368	1137*	1
*Selenomonas flueggei* ATCC 43531	Scaffolds/contigs	1 089.7	1 216*	1
*Selenomonas noxia* ATCC 43541	Scaffolds/contigs	1 364.8	1 296*	1
*Selenomonas sp* oral taxon 137 str F0430	Scaffolds/contigs	1 830.8	1 532	4
*Selenomonas sp* oral taxon 149 str 67H29BP	Scaffolds/contigs	1 252.5	1 258*	1
*Selenomonas sputigena* ATCC 35185	Complete	1 861.4	1 543	4
*Thermosinus carboxydivorans* Nor1	Scaffolds/contigs	1 898.8	1 549	7
*Veillonella atypica* ACS-049-V-Sch6	Scaffolds/contigs	1 512.8	1 369*	1
*Veillonella atypica* ACS-134-V-Col7a	Scaffolds/contigs	1 871.2	1 551	1
*Veillonella dispar* ATCC 17748	Scaffolds/contigs	1 870.5	1 551	3
*Veillonella parvula* ATCC 17745	Scaffolds/contigs	1 848.5	1 553	1
*Veillonella parvula* DSM 2008	Complete	1 859.5	1 551	4
*Veillonella sp* 3 1 44	Scaffolds/contigs	1 861.6	1 553	1
*Veillonella sp* 6 1 27	Scaffolds/contigs	1 862.2	1 551	1
*Veillonella sp* oral taxon 158 str F0412	Scaffolds/contigs	1 860.5	1 552	4
*Veillonella sp* oral taxon 780 str F0422	Scaffolds/contigs	1 877.1	1 550	4

The total number of identified 16S rRNA sequences is shown for each genome sequence. Length of highest scoring sequence and corresponding RNAmmer score is given. Default settings is to select the sequence with the highest RNAmmer score and a length between 1 400–1 800 bases. For this analysis the criteria were changed to a length range of 1 100–1 800, to include sequences from all genomes with 16S rRNA matches. Sequences with lengths shorter than the default acceptance threshold are marked with a “*”. Two organisms did not have any hits to the RNAmmer models, values of zero are marked by “−”.

The 16S rRNA tree (Figure 2) has been manually colored by genus, where multiple genomes per genus was available. The genomes show a general tendency to cluster within their taxonomical groups. Furthermore, the tree shows three main clusters with *Acidaminococcus* and *Selenomonas* as separate clusters (cluster II and III). The last cluster contains the genomes of *Veillonella*, *Megasphaera* and *Dialister*, all clustered in subgroups according to taxonomy. The clustering of genomes according to genera is expected since the taxonomic naming is based on 16S rRNA comparison [18]. It should be noted that the resulting trees shown here should be considered as preliminary classification.

### Genome Atlases (Structural DNA Atlas)

**Figure 3:**
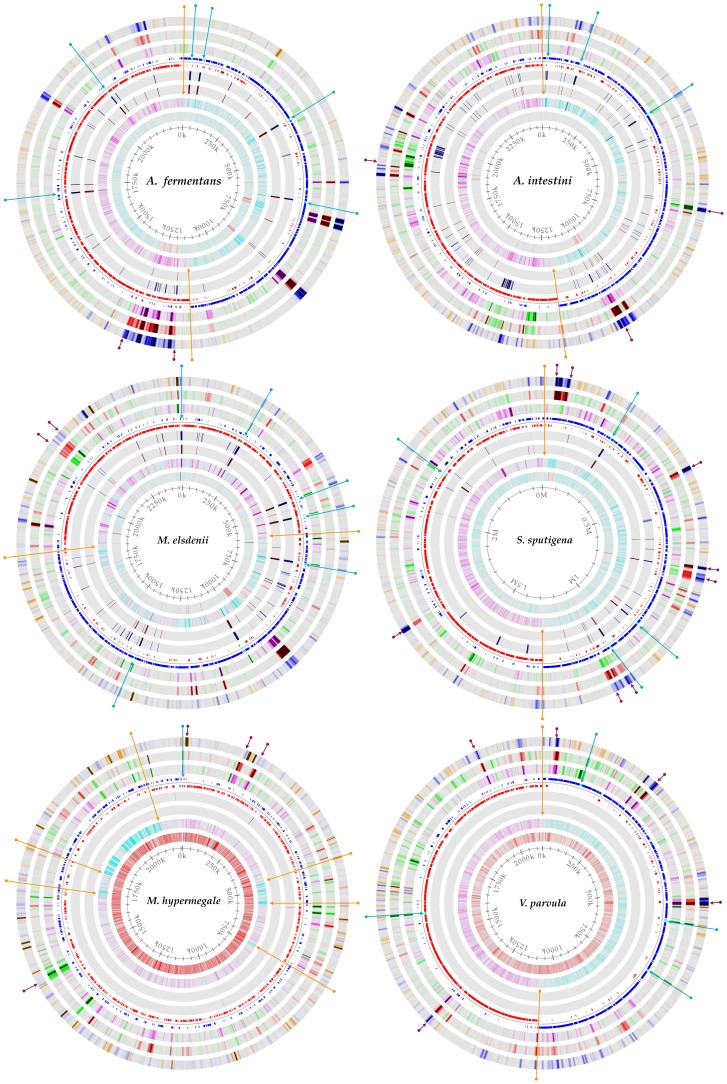
Genome atlases, DNA structures. A DNA structural atlas was generated for each of the 6 complete genomes. DNA, RNA and gene annotations are from the published GenBank data. Each lane of the circular atlas shows a different DNA feature. From the innermost circle: size of genome (axis), percent AT (red = high AT), GC skew (blue = most G’s), inverted and direct repeats (color = repeat), position preference, stacking energy and intrinsic curvature. Orange arrows indicate changes in the skew of G and C, which frequently indicate origin and terminus of replication. Blue arrows show the location of rRNA operons, as annotated in the GenBank file. Dark red arrows highlight areas of the genome that show significantly different DNA structures than the rest of the genome. A higher resolution pdf is available as a supplemental figure. A high resolution figure can be found as supplemental Figure S1.

Genome atlases were constructed for each of the 6 complete genomes using GenBank files generated by prodigalrunner(Table 1 and Figure 3, high resolution figure as supplemental Figure S1). The input to this analysis is a GenBank file containing one replicon of a genome (a single chromosome or plasmid, Figure 1, Step 3E). The analysis is performed using the program genomeAtlas, which is a collection of scripts that utilizes the GeneWiz program [6]. The genome atlas shows three types of information: base composition (AT content, GC skew), global repeats within the replicon (direct and inverted), and DNA structural properties (position preference, DNA stacking energy, and curvature). Genes (blue for leading and red for lagging strand), rRNAs and tRNAs are displayed as found in the GenBank annotation. The DNA is used for simple base count information includes AT content and GC skew. The atlas also shows a visual representation of structural properties of the DNA molecule (inverted and direct repeats, position preference [19], stacking energy [20] and intrinsic curvature [21], [22]). These different structures can potentially influence gene expression, likelihood of gene rearrangement and even evolutionary hotspots. The atlases in Figure 3 show a range of different DNA structure properties. Arrows and colors mark different important regions on each atlas (added to the atlases manually).

Mobile elements sometimes have different base composition, and can be indicated by areas of different curvature, stacking energy and position preference, compared to the chromosomal average (grey), as seen from the atlas of *Acidaminococcus fermentans*. Highly expressed regions are sometimes regions which will not easily condense around chromatin proteins (See atlas for *Acidaminococcus intestini* RyC-MR95, very low position preference, average stacking energy and position preference). Some regions are often associated with rRNA sequences and these patterns are also thought to correlate with high gene expression (See atlas for *Megasphaera elsdenii* DSM 20460, less negative stacking energy (red, melt easy) and low position preference (flexible)). Regions with high curvature and stacking energy indicate a strongly curved region with tendency to melt (See atlas for *Selenomonas sputigena* ATCC 35185). This structure might be involved in a special DNA structure, maybe where the chromosome attaches to the bacterial cell membrane. On the chromosome of *Veillonella parvula* DSM 2008 are several regions with high curvature, stacking energy and position preference, suggesting this region to be curved, rigid and easily melted. The genes in this region might be highly expressed but controlled by histone-like proteins that preferentially bind to curved DNA. The draft chromosome of *Megamonas hypermegale* ART12 1 is slightly different from the other atlases. For five of the six atlases in Figure 3, the GC skew indicates the location of the origin and terminus of replication, and changes from most G’s (blue) to more C’s (pink). For most bacterial genomes, G’s are biased toward the leading strand [23]. Note how the number of genes on leading/lagging strand changes along with the GC skew (more G’s, more minus strand genes). For the genome of *Megamonas hypermegale* ART12 1, the GC skew lane is a mixture of pink and blue, likely because this is a draft genome sequence. The genome is also highly AT rich (66%) and contains three regions with DNA structural patterns different from the rest of the genome.

### Amino Acid and Codon Usage

The input to the analysis of codon usage and bias in third codon position is a gene FASTA file (DNA). The amino acid usage can be performed on any set of proteins in FASTA format using aminoacidUsagePlot (Figure 1, Step 3D). Here, both analyses were run using the genes and proteins identified by prodigalrunner (Figure 1, Step 2C). The program basicgenomeanalysis calculates the bias in third position, codon and amino acid usage and the output is a text file containing the values along with a PDF file with plots. The bias is defined as −1 in the case of 100% A or T in third position, +1 is the case of 100% G or C.

**Figure 4:**
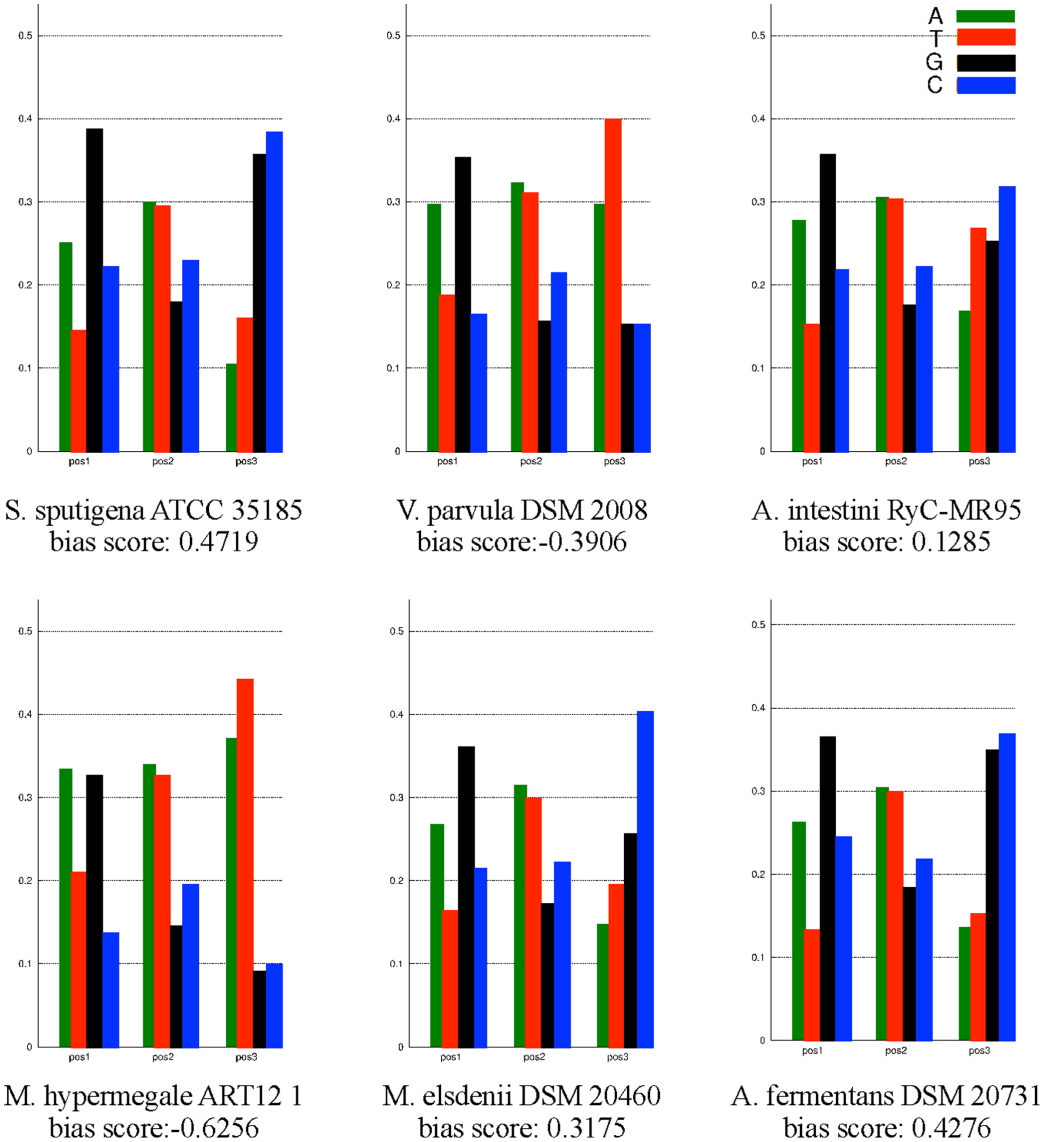
Bias in third position. The bias in third codon position is visualized for each of the 6 complete genomes. The bias was defined as −1 in the case of 100% A or T in third position, +1 is the case of 100% G or C.

The bias in third position was analyzed and visualized for the 6 complete genomes (Figure 4). The genomes of *V. parvula* DSM 2008 and *M. hypermegale* ART12 1 have a high bias towards A/T in third position (bias score, −0.3906 and −0.6256, respectively) and also a very high AT content (66% and 61%, respectively). The genomes of *S. sputigena* ATCC 35185 and *A. fermentans* DSM 20731, have low AT content and a bias towards G/C in third position (bias score, 0.4719 and 0.4276, respectively). *M. elsdenii* DSM 20460 and *A. intestini* RyC-MR95 have average AT content but *M. elsdenii* has a clear bias in third position towards C (bias score, 0.3175). This analysis shows the diversity of AT content between these genomes and also illustrates how AT content correlates with the nucleotide bias in third codon position.

**Figure 5:**
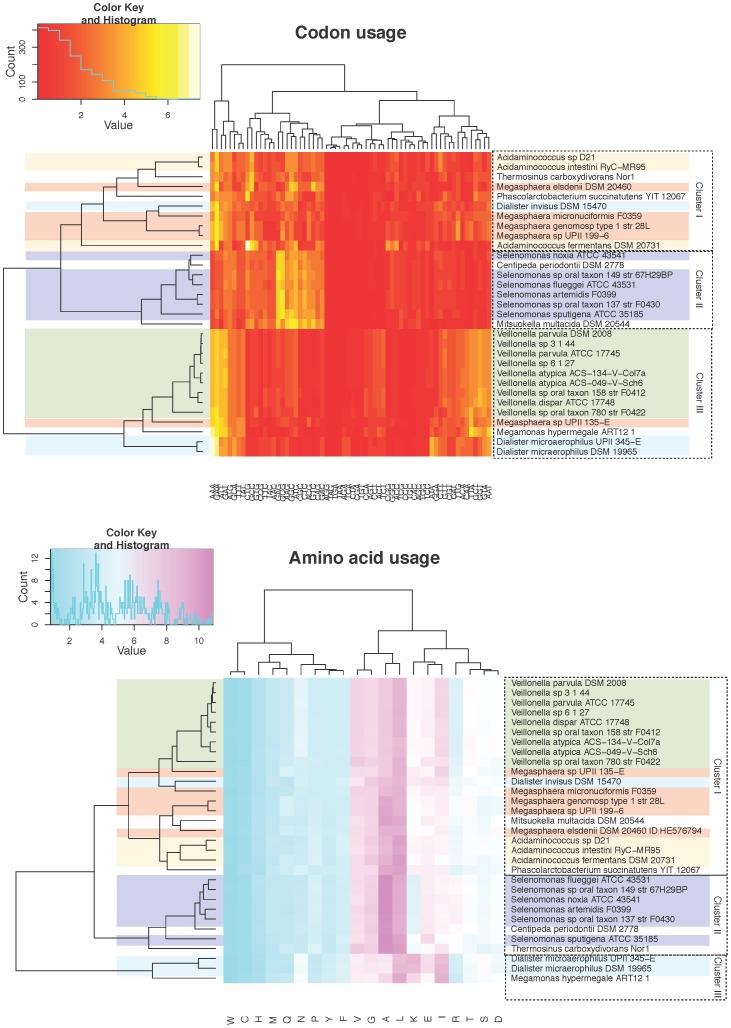
Amino acid and codon usage heatmaps. Amino acid and codon usage were for all 31 genomes calculated based on the genes identified by gene finding (Prodigal). The percentage of codon and amino acid usage was plotted in two heatmaps using R. The heatmaps were clustered in 2D, thus reordering the organisms and the amino acids/codon to show the shortest distance between them. Dendograms were draw for both and can be used to visualize the difference in usage between organisms.

The codon and amino acid usage was calculated for all 31 genomes and visualized in heatmaps created in R (Figure 5, genera colors were added manually). The genera of *Veillonella* and *Selenomonas* cluster together showing that each species have a unique use of both codons and amino acids. The genomes belonging to *Megasphaera*, *Acidaminococcus* and *Dialister* are less conserved, and do not consistently cluster together. These two trees show a different relationship than the 16S rRNA tree (Figure 2). The amino acid usage tree shows three main clusters with *Selenomonas* and *Dialister* forming their own clusters (cluster II and III). The last cluster (cluster I) consists of *Veillonella*, *Megasphaera* and *Acidaminococcus*. This is significantly different from the codon usage tree which creates a cluster consisting of *Veillonella* and *Dialister* with a single *Megasphaera* genome (cluster III), another cluster of *Selenomonas*(cluster II) and the last cluster of *Megasphaera* and *Acidaminococcus* (cluster I). None of the two methods makes a consistent clustering of the *Megasphaera* genomes as the 16S rRNA tree. In accordance, none of the three trees show the same general clusters, however they all manage to cluster closely related genomes, with the single exception of *Megasphaera*.

### Proteome Comparisons Using BLAST

For this analysis, proteomes were constructed for all 31 genomes using prodigalrunner for genefinding. Presented here are two different types of proteome comparisons, both based on the BLAST algorithm (Basic Local Alignment Search Tool) [24], [25]. The first method is a BLAST matrix and shows a pairwise proteome comparison by using BLAST to identify whether two proteins are shared between genomes [26]. Two proteins are considered to be in the same family if 50% of the alignment consists of identical matches and the length of the alignment is 50% of the longest gene. The main part of the matrix consists of pairwise genome comparisons; with fractions of shared proteins shaded in green (more green, more protein families shared). The row that would reflect self-comparison indicates internal homologs (internal paralogs, shaded red) which are defined as a significant hit within a genome to a protein other than the query protein itself.

The program performing this analysis is called blastmatrix and the input is an XML file (Figure 1, Step 3C). This file is created by the program makebmdest by inputting the name of a directory containing protein files. This program takes all the protein FASTA files in a given directory, extracts relevant information and formats it into an XML file which is read by the *blastmatrix* program. The protein FASTA file can be obtained by extracting proteins from a GenBank file (using saco_extract) or by using the Prodigal genefinder (extract DNA from GenBank, saco_convert, and find genes using prodigalrunner). A BLAST matrix comparison of the 31 *Negativicutes* genomes was calculated on the CMG-biotools system, using 4 processors (calculation time was 9 hours).

**Figure 6:**
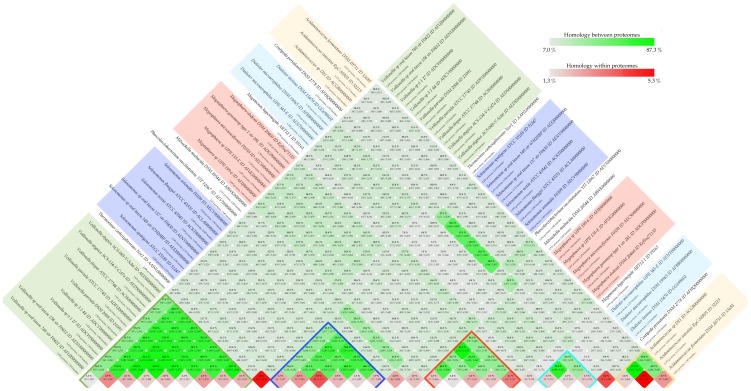
BLAST matrix. An all against all protein comparison was performed using BLAST to define homologs. A BLAST hit is considered significant if 50% of the alignment consists of identical matches and the length of the alignment is 50% of the longest gene. Internal homology (paralogs) is defined as proteins within a genome matching the same 50–50 requirement as for between-proteome comparisons. Self-matches are here ignored. A comparison of 31 *Negativicutes* genomes was performed on the CMG-biotools system (9 hours). A high resolution figure can be found as supplemental Figure S2.

The BLAST matrix (Figure 6, high resolution figure as supplemental Figure S2) illustrates that the conservation between genomes is generally higher within a genus than between genera (for example *Selenomonas*, 53–57%, and *Megasphaera*, 33–81%). The *Selenomonas* strains also show a high similarity to the genome of *C. periodontii* DSM 2778, while the *Megasphaera* genus shows no higher similarity to other genera. For both the genomes of *Acidaminococcus* and *Dialister*, the similarity is varied with one comparison being very similar and the others not (31–45%). Within the *Veillonella* genus, the conservation is 64–84% with the exception of *Veillonella* species oral taxon 780 str F0422 (conservation 36–38% to other *Veillonella*). In comparison, a study performed on genomes from the *Vibrionaceae* family showed that different strains of *Vibrio cholerae* share between 70–80% proteins while the similarity to organisms outside the species ranged from 30–45% [27]. From that same study, the internal homology (red squares) ranges from 1.3–5.3%. Other studies, such as a study on *Vibrionaceae* have shown numbers ranging from 1.8–5%. Another study analyzed the similarity between *Enterobacteriaceae* genomes, and found a 76–98.8% similarity between 7 genomes of *Escherichia coli* [28] The same study showed an internal homology of approximately 0.3–3% for the 7 *Escherichia coli*.

**Figure 7:**
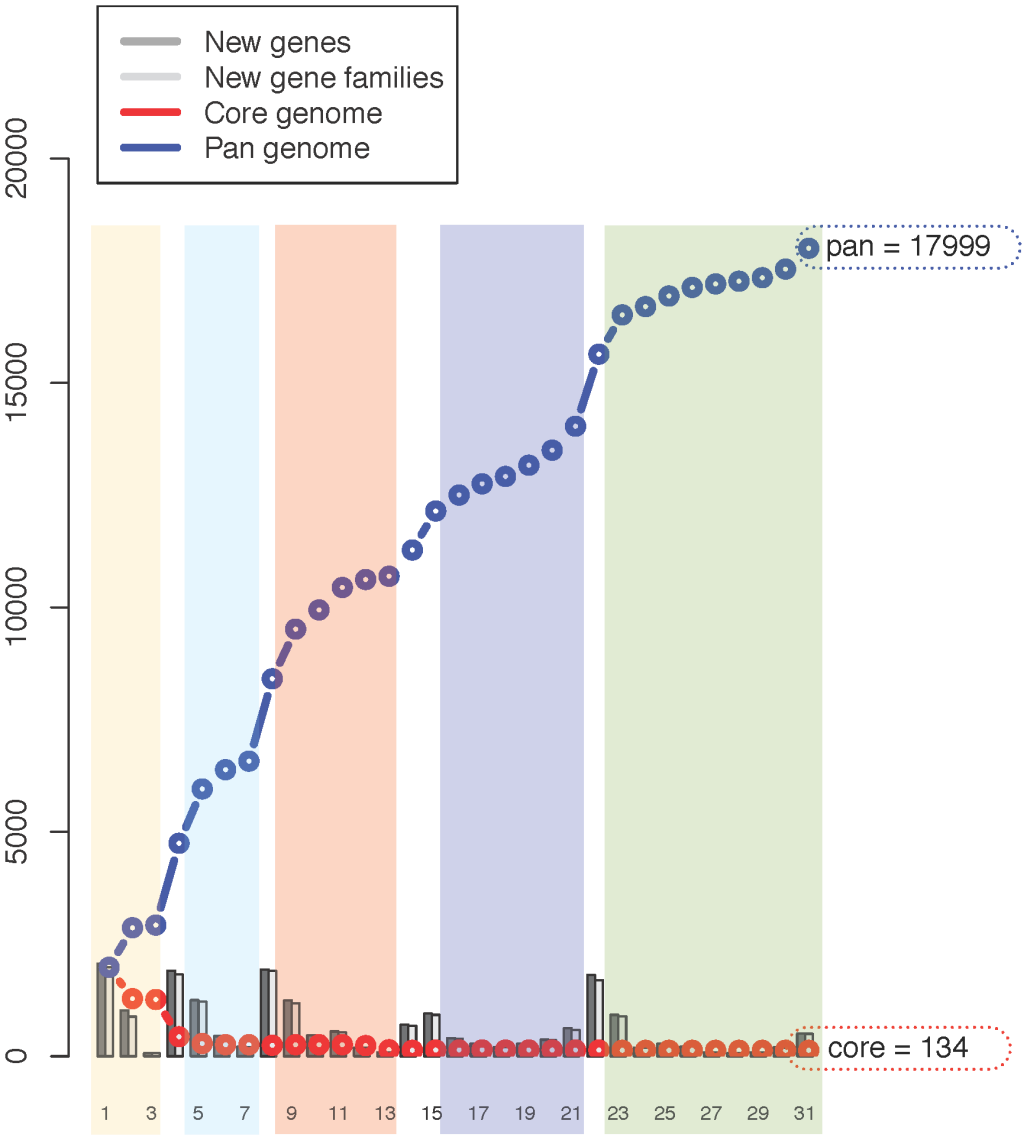
Core and pan genome using BLAST. A pan- and core-genome calculation was performed using BLAST. A BLAST cutoff of 50% identity and 50% coverage of the longest gene was used. If two proteins within a genome matched according to the 50/50% cutoff, they were clustered into one protein family. Protein families were extended via single linkage clustering. If a protein family includes proteins from all genomes in the comparison, the family is a core protein family.

The second method looks at the cumulative set of all genes, shared across genomes (pan-genome) and the conserved set of gene families across all genomes (core-genome) [29]. The pan- and core-genomes are theoretical representations of a collective protein pool and a conserved protein pool, respectively. When a protein type is found in all genomes in a collection, it is called a core gene of this collection. Here this is implemented in a pan- and core-genome plot (Figure 7) where sequences are compared using BLAST and the 50/50% cutoff described above. As the clusters grow to more than two members, single linkage clustering is used to assign a new sequence to a group. The program performing this analysis is called pancoreplot and the input is a tab separated text file representing a number of FASTA files containing amino acid sequences (Figure 1, Step 3C). For this analysis, the input files and directories are the same as described for the BLAST matrix.

For the first genome, the pan and core are identical, and the core becomes smaller with the addition of a second genome, as genes in this pool now need to be found in both genomes. If a gene from the core is not found in a new genome it is removed from the core, and is then only part of the pan-genome. The pan-genome is the entire gene pool and as such includes the core genome. The order of the genomes can change the course of the graph, but the final shared gene pool (core and pan-genome) will be the same.

A pan- and core-genome plot analysis was performed for all 31 genomes (Figure 7). The final core genome was found to be 134 gene families and the pan genome contains 17 999 gene families. For an average proteome size of around 1 900 within the *Negativicutes*, a core genome of 134 is relatively small. Using the output data from the pan- and core-genome it was possible to analyze gene overlaps and intersections of the dataset. The core genome of the *Veillonella* genomes is 936 protein families, less than half of the average number of genes in these genomes. Of these families, nly 210 are not found in any of the other genomes (complimentary) while 802 families are not found in the core of the other genomes (“compinter”). The pan-genome of the 31 genomes is 17 999 families, indicating a large diversity and many accessory genes in this class. Compared to similar analyses for genomes of the *Vibrionaceae* family, pan- and core-genome sizes was 20 200 and 1 000 respectively [27]. The *V. cholerae* genomes have a core genome of 2 500, more than 60% of the average size of these genomes, 4 000 genes [27].

## Materials and Methods

### The CMG-biotools

CMG-biotools is a modified setup of the publicly available Xubuntu 10.10 (www.xubuntu.org/get) operating system. Xubuntu is a community developed operating system that is well-suited for laptops and desktops. It natively contains all applications from word processing and email applications to web server software and programming tools and is part of the Ubuntu project, published under the GPL (GNU General Public License). A number of bioinformatic tools have been added to the system to allow for analysis of microbial genome sequence data and is here called “CMG-biotools”. CMG-biotools is an installable operating system (disc image,.iso format). By installing the software, the user accepts the terms of the license and agreements.

The CMG-biotools operating system can be installed on a local computer or on a virtual computer application, such as VirtualBox (www.virtualbox.org). A standard installation should take less than 25 minutes. The functionalities of CMG-biotools consists of a series of compiled executables, Perl, Python and bash scripts contained in a folder on the system (/usr/biotools/). These scripts can be modified according to the individual licenses of the programs (See.*LICENSE* files for this information). The CMG-biotools system is made to run on a local laptop and uses one processor by default. The computationally heavy programs, blastmatrix and pancoreplot, have built-in options (-cpu) that allows the user to increase the number of processors if available.

### Download

The installable disk image file (.*iso*) containing CMG-biotools is available from the webpage (www.cbs.dtu.dk/staff/dave/CMGtools/). The tutorials for the courses taught on this platform are available from the same webpage. The system has been tested using VirtualBox, a free virtual computer application, on Windows and Mac operating systems (www.virtualbox.org).

### Programs

#### Data collection

The getgbk.pl script uses the Entrez E-utils programmatic interface made available by the NCBI to fetch sequence data. The script allows searching within the NCBI nuccore or the new bioproject databases using Genbank Accession identifiers or project identifiers respectively. Records identified in *bioprojects* can be filtered to only fetch matches in RefSeq or GenBank. Extraction of DNA from GenBank format is done using saco_convert [12], which locates the DNA sequences in the GenBank data labeled “ORIGIN” and prints the data in FASTA format. Extraction of translated coding sequences from GenBank is done using saco_extract [12]. This program accesses the GenBank data labeled “translation”, extracts the sequences and prints them as FASTA format along with the gene identifier, also obtained from the GenBank file. Some GenBank files do not have annotated protein sequences and from these the extraction procedure will not work. In such cases, genefinding should be performed. The input arguments to the saco programs describes input and output file formats, where the first indicates the input file format (for instance GenBank) and the second the output format (for instance FASTA).

#### Phylogenetic analysis

The RNAmmer [14] program is used for the localization of rRNA sequences in genomic DNA (FASTA format). DNA is extracted from GenBank files using saco_convert and stored in FASTA format (Figure 1, Step 2B). The program uses HMM models to search a DNA sequence for sequences with significant similarity to models of rRNA sequences. Models are included for 5S, 16S and 23S rRNA for bacterial genomes (options TSU, SSU and LSU respectively). For the examples in this paper, each genome sequence was compared to the models for 16S rRNA only. Each sequence is searched and possible rRNA sequences are stored as FASTA formatted DNA sequences. The highest scoring sequence with acceptable length (between 1 400 and 1 800) is extracted from each genome (select16SrRNA) and stored in a FASTA formatted DNA file. It is also possible to use all predicted sequence in stead of selecting the highest scoring one. Some genomes have multiple 16S rRNA sequences and they might yield slightly different phylogenetic relationships. One sequence from each genome is compared in a multiple alignment using ClustalW [15] and the resulting alignment is used to construct a distance tree using 1 000 re-samplings. The tree is visualized using njplot [17].

#### Genome atlases (structural DNA atlas)

The genome atlas presented here is an implementation of the atlas presented earlier by Jensen et al. 1999 [4], [6]. Below is a short description of each of the parameters shown in the DNA atlases. Color scales for all parameters follow the same system. The DNA sequence is read and an output file is generated for the various calculated parameters. For each nucleotide in the genome a numerical value is calculated. This file is then read by the GeneWiz program, which calculates the average and standard deviation for each parameter, if the average value of the window is more than 3 standard deviations on either side of the overall average the window is maximally colored. In order to plot the data on a circular map a “window size” is used for longer genomes, which effectively smooths the data for better graphics. For the parameters *Stacking Energy*, *Position Preference* and *Intrinsic Curvature*, the window is 0.002×genome length. The window is 0.001×genome length for *Percent AT* and *GC skew*. Each of these are calculated separately, wrapped into a pipeline and visualized in a circular plot, called an atlas. The gene annotations are taken directly from a GenBank coding regions; if no such information is found the *CDS−/+* lanes will be blank. The following lists explanations to each of the lanes in a genome atlas: **Percent AT** is the percent of A’s and T’s in the genome. **GC skew** is calculated as *((G-C)/(G+C))*, with a window size of 10 000 bp and is useful for determining the origin and terminus of replication [30], [31]. **Global Direct Repeats** and **Global Inverted Repeats** refer to a sequence that is present in at least two copies on the same or opposite strands, respectively. **Intrinsic Curvature** is a measure of DNA curvature and is calculated using the CURVATURE program [21], [22]. The values are scaled from 0 (e.g. no curvature) to 1, which is the curvature of DNA when wrapped around the nucleosome. **Stacking Energy** is derived from the dinucleotide values provided by Ornstein et al [20]. The scale is in kcal/mol, and the dinucleotide values range from −3.82 kcal/mol (will unstack easily) to −14.59 kcal/mol (difficult to unstack). A positive peak in base-stacking (i.e., numbers closer to zero) reflects regions of the helix which would de-stack or melt more readily. Conversely, minima (larger negative numbers) in this plot would represent more stable regions of the chromosome. **Position Preference** is a measure of preferential location of sequences within nucleosomal core sequences [19]. The trinucleotide values range from essentially zero (0.003, presumably more flexible), to 0.28 (considered rigid). Since very few of the trinucleotide have values close to zero (e.g. little preference for nucleosome positioning), this measure is considered to be more sensitive towards the low (“flexible”) end of the scale.

#### Gene finding

Gene finding is performed using the program Prodigal [13]. The program is wrapped into a formatting program called prodigalrunner. The program reformats the raw output of Prodigal to FASTA formatted open reading frames, DNA and amino acids, along with a draft of a GenBank file and a raw general feature formatted file, a.gff file. The Prodigal program allows for different parameter modifications, including training (prodigalrunner -t <organism>) of the gene finder using given data. This feature increases the computation time of the algorithm, but for less known organisms this feature might improve gene finding. It should be noted that the default behavior when encountering N’s is not changed - the program treats runs of N’s as masked sequence and does not build genes across them. The CMG-Biotools system also comes with the native Prodigal program, which can be used as published [13].

#### Amino acid and codon usage

The amino acid and codon usage is calculated using BioPerl modules [32], and is a simple calculation of the fraction of each amino acid or codon count of the total count of amino acids or codons. The bias in third position is found by counting the number of each base on each position in each codon, divided by the total number of codons. The bias in the third position between *G/C* and *A/T* was then calculated as *sum(GC)-sum(AT)*, so that 100% GC in third codon position is +1 and −1 for 100% AT. The plots are made using Perl and Gnuplot.

#### Proteome comparisons using BLAST

The BLAST matrix is a visual presentation of a pairwise proteome comparison using BLAST (Basic Local Alignment Tool) [26]. All sequences are compared to each other and a BLAST hit is significant when 50% of the alignment is identical matches and the length of the alignment is 50% of the longest gene in the comparison. If two sequences are similar according to the cutoff, they are collected in one “protein family”. For the comparison of two genomes, protein families are built through single linkage, so that each shared connection must be between sequences from different genomes (shaded green). Paralogs are traditionally defined as a gene which has undergone duplication before speciation; in the BLAST matrix, an internal hit significantly similar to the query protein is grouped into the same gene family. The bottom row of the matrix shows the number of proteins that have homologous hits within the proteome itself (shaded red). The color scales are set automatically from the highest to lowest value observed, but can be changed manually. The procedure is implemented in the program blastmatrix, which takes a XML formatted input file. The input file is created by the program makebmdest.

The pan- and core-genome plot is a different use of BLAST for comparing proteomes (using the 50/50 cutoff as described above). The core-genome consists of protein families with representatives found in all investigated genomes. The pan-genome is the entire set of protein families from all genomes in the comparison. The first genome in the analysis has a core-genome equal to the pan-genome. The addition of an second genome reduces the core-genome of the two genomes and increases the pan-genome. Each sequence of a new genome is compared to a representative from each of the existing gene-families. If the new sequence matches, the family is a core-family, if the sequence does not match a family it becomes a new protein family. When all new sequences have been compared to existing gene-families, core families that did not have a representative in the latest added genome are removed from the core-genome of the genome comparison. The change in the pan- and core-genome is followed as two lines (blue and red, respectively). The number of new proteins, along with how many new protein families that corresponds to, is indicated as gray bars on the plot. The program (pancoreplot), produces a plot and a table which can be used to look up the underlying values of the plot.

The pan- and core-genome calculations can be used to extract subsets of genes for different genome sets. The program that implements this is called specificGenes and works on the BLAST output from the pancoreplot program. The procedure is based on mathematical set theory and works with intersections, unions and complementary genesets. Each genome is treated as a set and the intersection is the gene families that two or more sets have “in common”. The intersection of genome A and B, is the set of all gene families which are found in both A and B. The union of two or more sets refers to the gene families which are found in either genome A or B. Calculating the complimentary families of a genome refers to the set of all families which are members of A but not members of B. In the comparative genomic analysis, the sets usually consists of more than one genome, such as the intersection of genome A, B and C while not found (complimentary) in genome D, E and F. This will give families that are found in A, B and C but not found in any of D, E or F. It is also possible to calculate the situation where families are found in A, B and C but not found in the intersection of D, E and F, this is referred to as the “compinter”. For more details, see the CMG-biotools manual.

## Supporting Information

Figure S1 **Genome atlases, DNA structures (** **Figure 3** **at High-Resolution).**
(PDF)

Figure S2 **BLAST matrix (** **Figure 6** **at High-Resolution).**
(PDF)

## References

[1] Casali N, Nikolayevskyy V, Balabanova Y, Ignatyeva O, Kontsevaya I, et al.. (2012) Microevolution of extensively drug-resistant tuberculosis in Russia. Genome Research : 735–745. 10.1101/gr.128678.111 PMC3317155 22294518

[2] Fleischmann RD, Adams MD, White O, Clayton RA, Kirkness EF, et al (1995) Whole-genome random sequencing and assembly of *Haemophilus inuenza* Rd. Science 269: 496–512. 7542800 10.1126/science.7542800

[3] Fraser CM, Gocayne JD, White O, Adams MD, Clayton RA, et al (1995) The minimal gene complement of *Mycoplasma genitalium* . Science 270: 397–403. 7569993 10.1126/science.270.5235.397

[4] Jensen LJ, Friis C, Ussery DW (1999) Three views of microbial genomes. Research in Microbiology 150: 773–777. 10673014 10.1016/s0923-2508(99)00116-3

[5] Friis C, Jensen LJ, Ussery DW (2000) Visualization of pathogenicity regions in bacteria. Genetica 108: 47–51. 11145420 10.1023/a:1004091626474

[6] Pedersen aG, Jensen LJ, Brunak S, Staerfeldt HH, Ussery DW (2000) A DNA structural atlas for *Escherichia coli* . Journal of Molecular Biology 299: 907930. 10.1006/jmbi.2000.3787 10843847

[7] Snipen L, Almø y T, Ussery DW (2009) Microbial comparative pan-genomics using binomial mixture models. BMC Genomics 10: 385. 19691844 10.1186/1471-2164-10-385 PMC2907702

[8] Pilo P, Frey J (2011) Bacillus anthracis: Molecular taxonomy, population genetics, phylogeny and patho-evolution. Infection, Genetics and Evolution 11: 12181224. 10.1016/j.meegid.2011.05.013 21640849

[9] Tettelin H, Masignani V, Cieslewicz MJ, Donati C, Medini D, et al (2005) Genome analysis of multiple pathogenic isolates of *Streptococcus agalactiae*: implications for the microbial “pangenome”. Proceedings of the National Academy of Sciences of the United States of America 102: 1395013955. 10.1073/pnas.0506758102 PMC1216834 16172379

[10] DistroWatch (accessed 17/09/2012). http://distrowatch.com/dwres.php?resource=popularity

[11] Benson Da, Karsch-Mizrachi I, Lipman DJ, Ostell J, Sayers EW (2011) GenBank. Nucleic Acids Research 39: D32–D37. 21071399 10.1093/nar/gkq1079 PMC3013681

[12] Jensen L, Knudsen S (1999) Automatic discovery of regulatory patterns in promoter regions based on whole cell expression data and functional annotation. Bioinformatics 16: 326–333. 10.1093/bioinformatics/16.4.326 10869030

[13] Hyatt D, Chen GL, Locascio PF, Land ML, Larimer FW, et al (2010) Prodigal: prokaryotic gene recognition and translation initiation site identification. BMC Bioinformatics 11: 119. 20211023 10.1186/1471-2105-11-119 PMC2848648

[14] Lagesen K, Hallin P, Rø dland EA, Staerfeldt HH, rn Rognes T, et al (2007) RNAmmer: consistent and rapid annotation of ribosomal RNA genes. Nucleic Acids Research 35: 3100–3108. 17452365 10.1093/nar/gkm160 PMC1888812

[15] Larkin Ma, Blackshields G, Brown NP, Chenna R, McGettigan Pa, et al (2007) Clustal W and Clustal X version 2.0. Bioinformatics 23: 2947–2948. 17846036 10.1093/bioinformatics/btm404

[16] Felsenstein J (1985) Confidence limits on phylegenies an approach using bootstrap. Evolution 39: 783–791. 28561359 10.1111/j.1558-5646.1985.tb00420.x

[17] Perrière G, Gouy M (1996) WWW-query: an on-line retrieval system for biological sequence banks. Biochimie 78: 364–369. 8905155 10.1016/0300-9084(96)84768-7

[18] Woese CR, Fox GE (1977) Phylogenetic structure of the prokaryotic domain: the primary kingdoms. Proceedings of the National Academy of Sciences of the United States of America 74: 5088–5090. 270744 10.1073/pnas.74.11.5088 PMC432104

[19] Satchwell SC, Drew HR, Travers AA (1986) Sequence periodicities in chicken nucleosome core DNA. Journal of Molecular Biology 191: 659–675. 3806678 10.1016/0022-2836(86)90452-3

[20] Ornstein RL, Rein R, Breen DL, Macelroy RD (1978) An optimized potential function for the calculation of nucleic acid interaction energies. I - Base stacking. Biopolymers 17: 2341–2360. 24624489 10.1002/bip.1978.360171005

[21] Shpigelman ES, Trifonov EN, Bolshoy A (1993) CURVATURE: software for the analysis of curved DNA. Computer Applications in the Biosciences CABIOS 9: 435–440. 8402210 10.1093/bioinformatics/9.4.435

[22] Bolshoy A, McNamara P, Harrington RE, Trifonov EN (1991) Curved DNA without A-A: experimental estimation of all 16 DNA wedge angles. Proceedings of the National Academy of Sciences of the United States of America 88: 2312–3216. 2006170 10.1073/pnas.88.6.2312 PMC51221

[23] Marín A, Xia X (2008) GC skew in protein-coding genes between the leading and lagging strands in bacterial genomes: new substitution models incorporating strand bias. Journal of Theoretical Biology 253: 508–513. 18486155 10.1016/j.jtbi.2008.04.004

[24] Altschul SS, Gish W, Miller W, Myers EE, Lipman D, et al (1990) Basic Local Alignment Search Tool. Journal of Molecular Biology 215: 403–410. 2231712 10.1016/S0022-2836(05)80360-2

[25] Altschul SF, Madden TL, Schäffer AA, Zhang J, Zhang Z, et al (1997) Gapped BLAST and PSI-BLAST: a new generation of protein database search programs. Nucleic Acids Research 25: 3389–3402. 9254694 10.1093/nar/25.17.3389 PMC146917

[26] Binnewies TT, Hallin PF, Staerfeldt HH, Ussery DW (2005) Genome Update: proteome comparisons. Microbiology (Reading, England) 151: 1–4. 10.1099/mic.0.27760-0 15632419

[27] Vesth T, Wassenaar TM, Hallin PF, Snipen L, Lagesen K, et al (2010) On the Origins of a Vibrio Species. Microbial Ecology 59: 1–13. 19830476 10.1007/s00248-009-9596-7 PMC2807590

[28] Willenbrock H, Petersen A, Sekse C, Kiil K, Wasteson Y, et al (2006) Design of a seven-genome *Escherichia coli* microarray for comparative genomic profiling. Journal of Bacteriology 188: 7713–7721. 16963574 10.1128/JB.01043-06 PMC1636325

[29] Klockgether J, Würdemann D, Wiehlmann L, Binnewies TT, Ussery DW, et al. (2008) Genome Diversity of *Pseudomonas aeruginosa*. Chapter 2 in Pseudomonas: Genomics and Molecular Biology, (Edited by: Pierre Cornelis, Caister Academic Press).

[30] Lobry JR (1996) Asymmetric substitution patterns in the two DNA strands of bacteria. Molecular Biology and Evolution 13: 660–665. 8676740 10.1093/oxfordjournals.molbev.a025626

[31] Worning P, Jensen LJ, Hallin PF, Staerfeldt HH, Ussery DW (2006) Origin of replication in circular prokaryotic chromosomes. Environmental Microbiology 8: 353–361. 16423021 10.1111/j.1462-2920.2005.00917.x

[32] Stajich JE, Block D, Boulez K, Brenner SE, Chervitz SA, et al (2002) The Bioperl toolkit: Perl modules for the life sciences. Genome Research 12: 1611–1618. 12368254 10.1101/gr.361602 PMC187536

