# Supplementary figures and images for: CMG-Biotools, a Free Workbench for Basic Comparative Microbial Genomics
Source: PLoS One. 2013 Apr 5;8(4):e60120. doi: 10.1371/journal.pone.0060120 (PMC3618517; doi:10.1371/journal.pone.0060120)

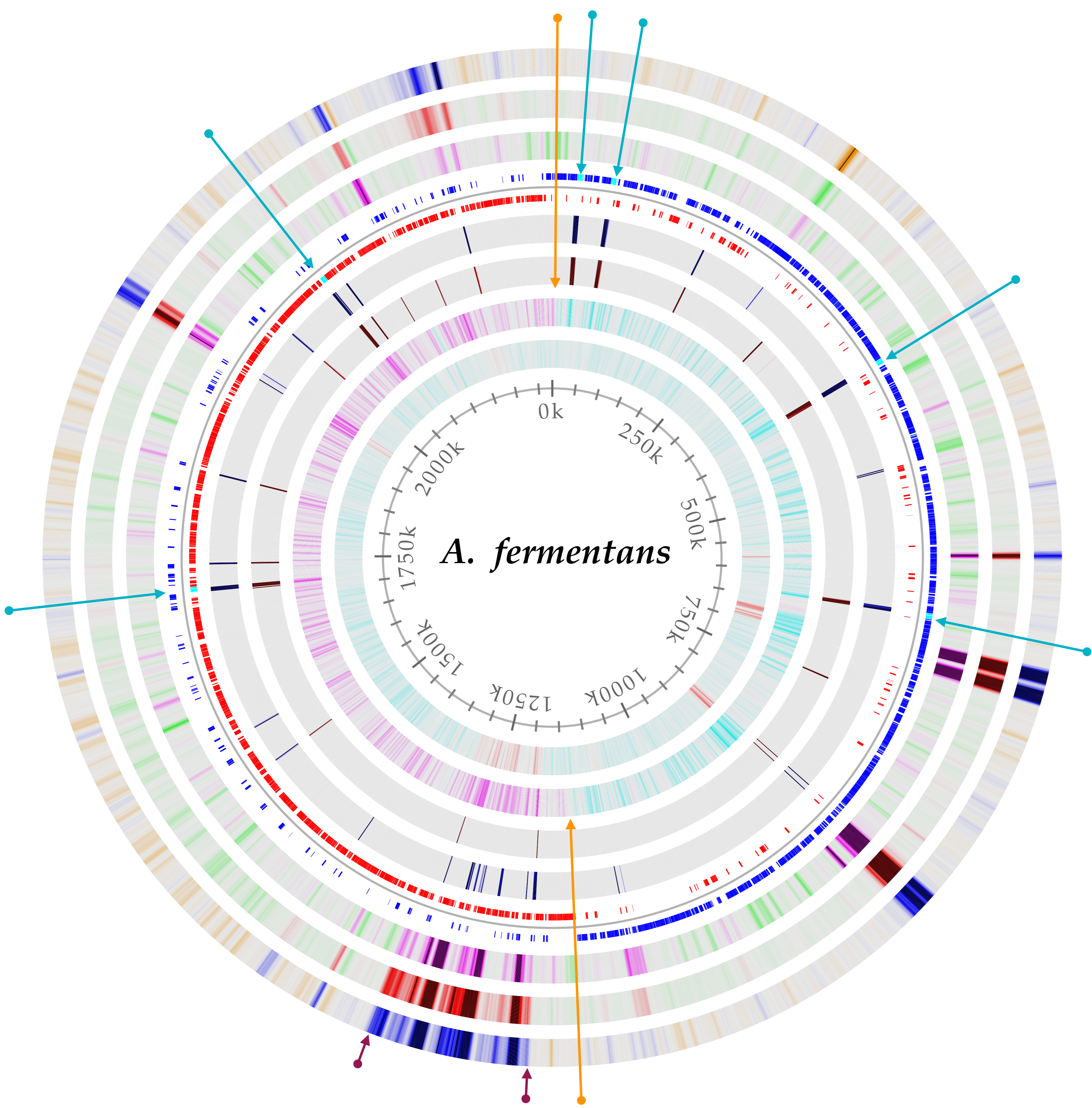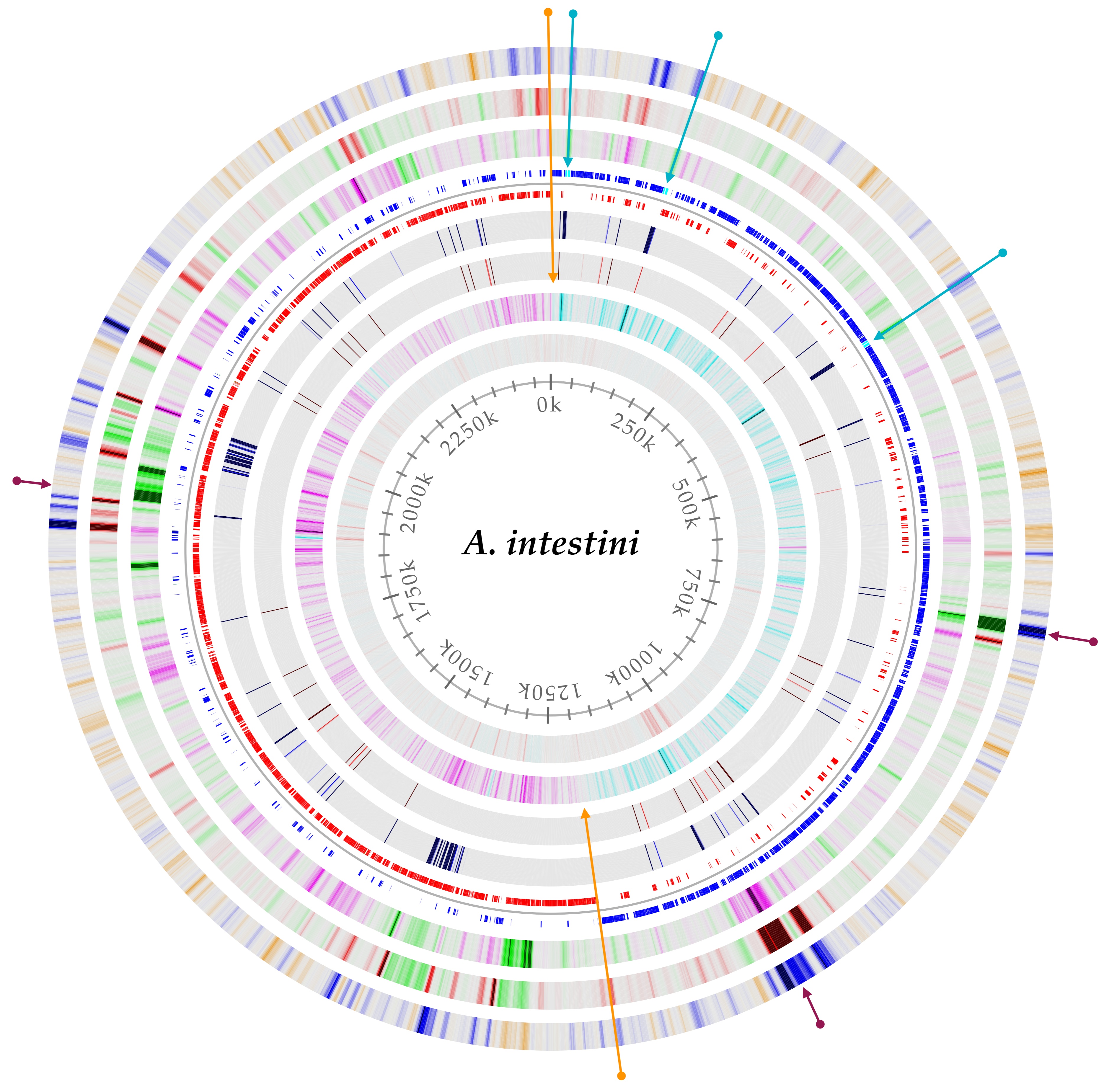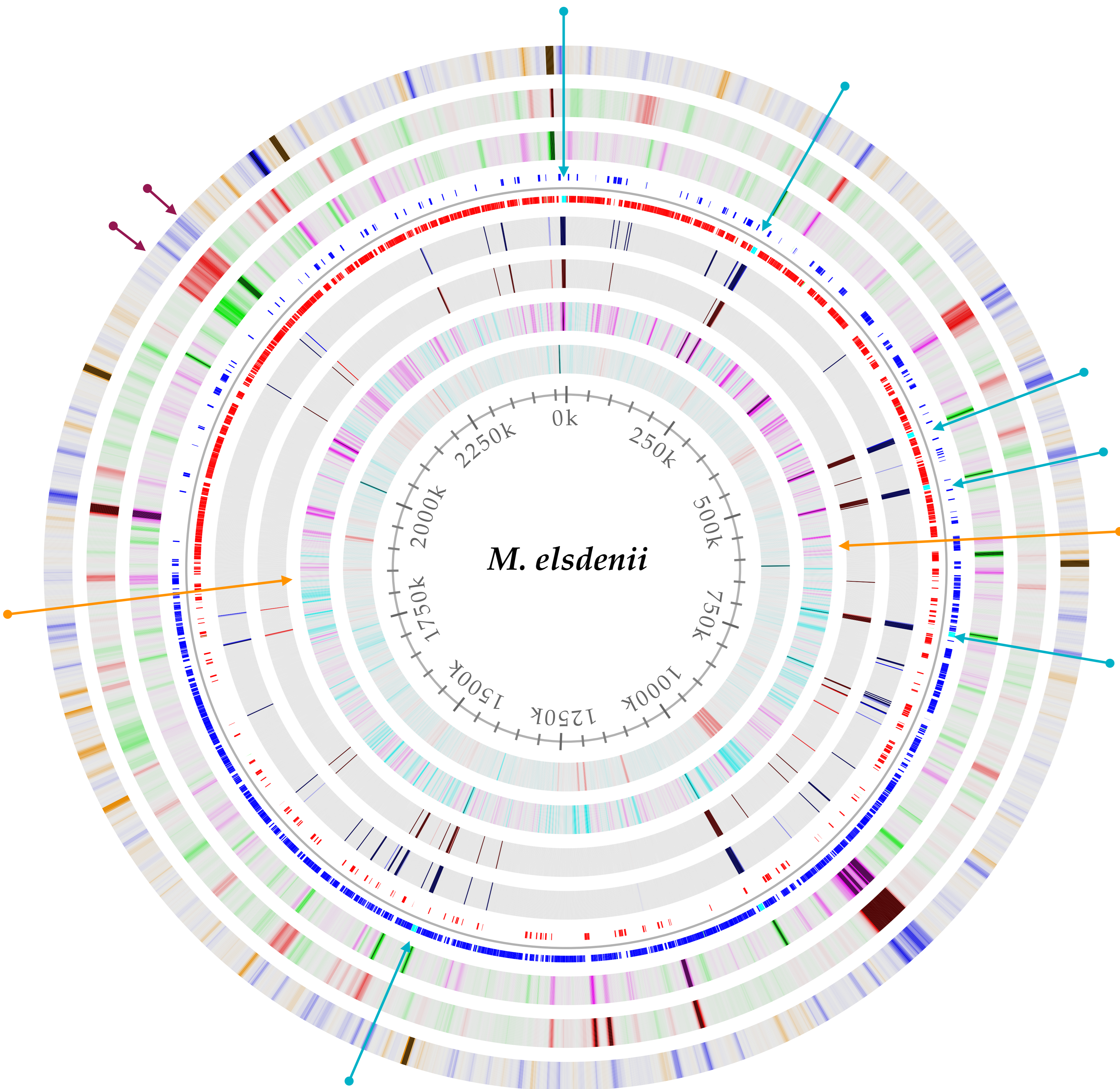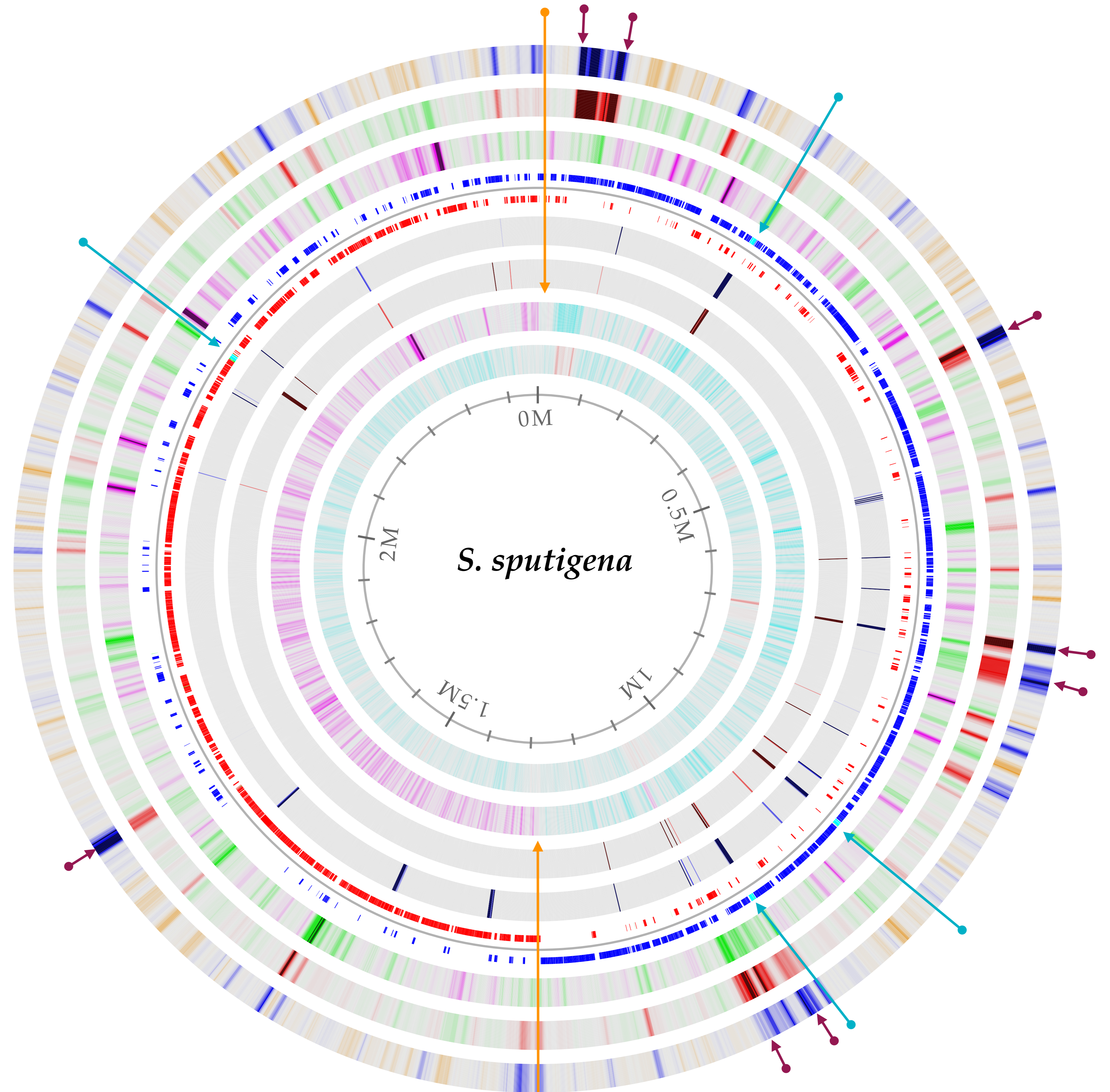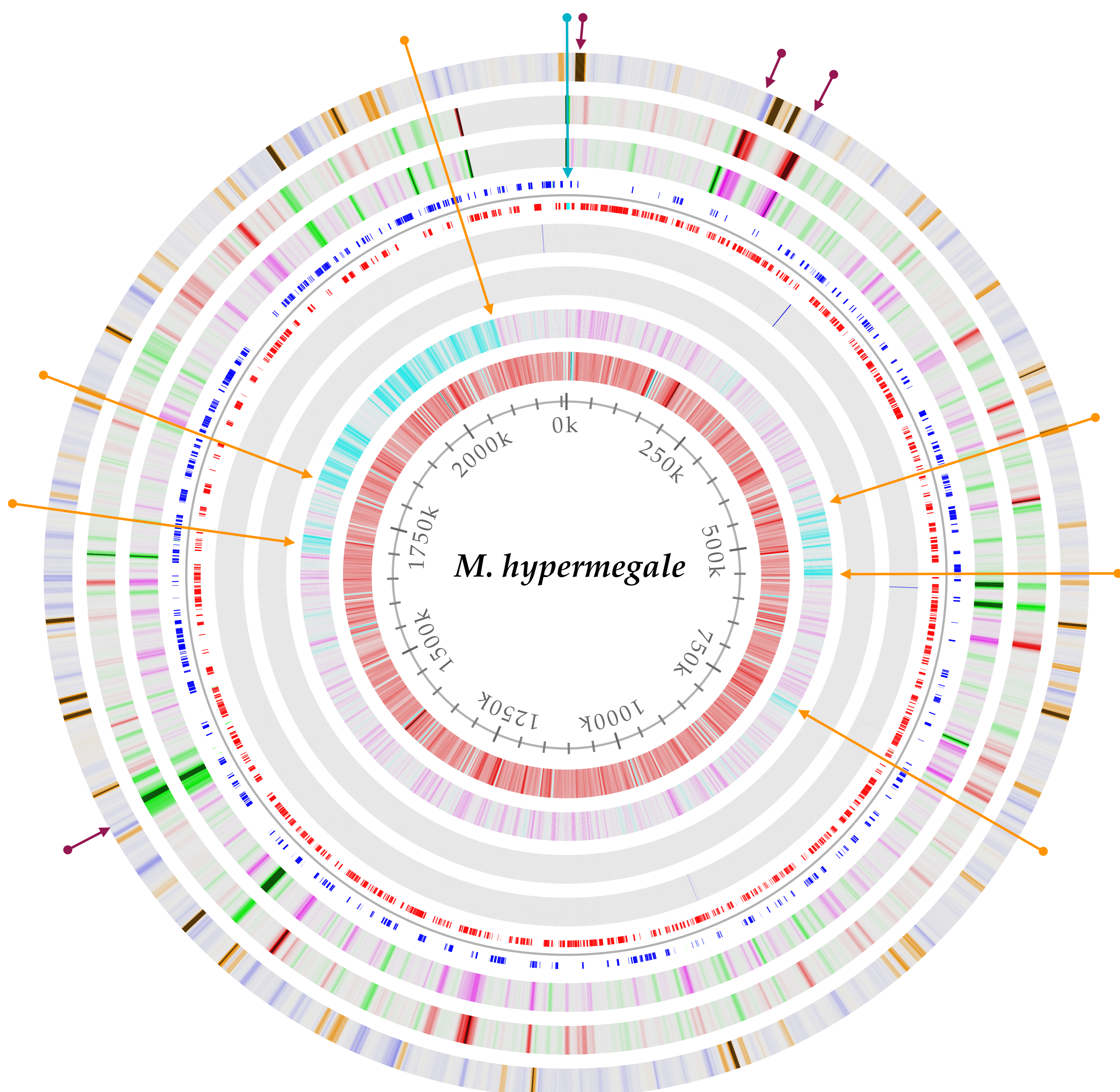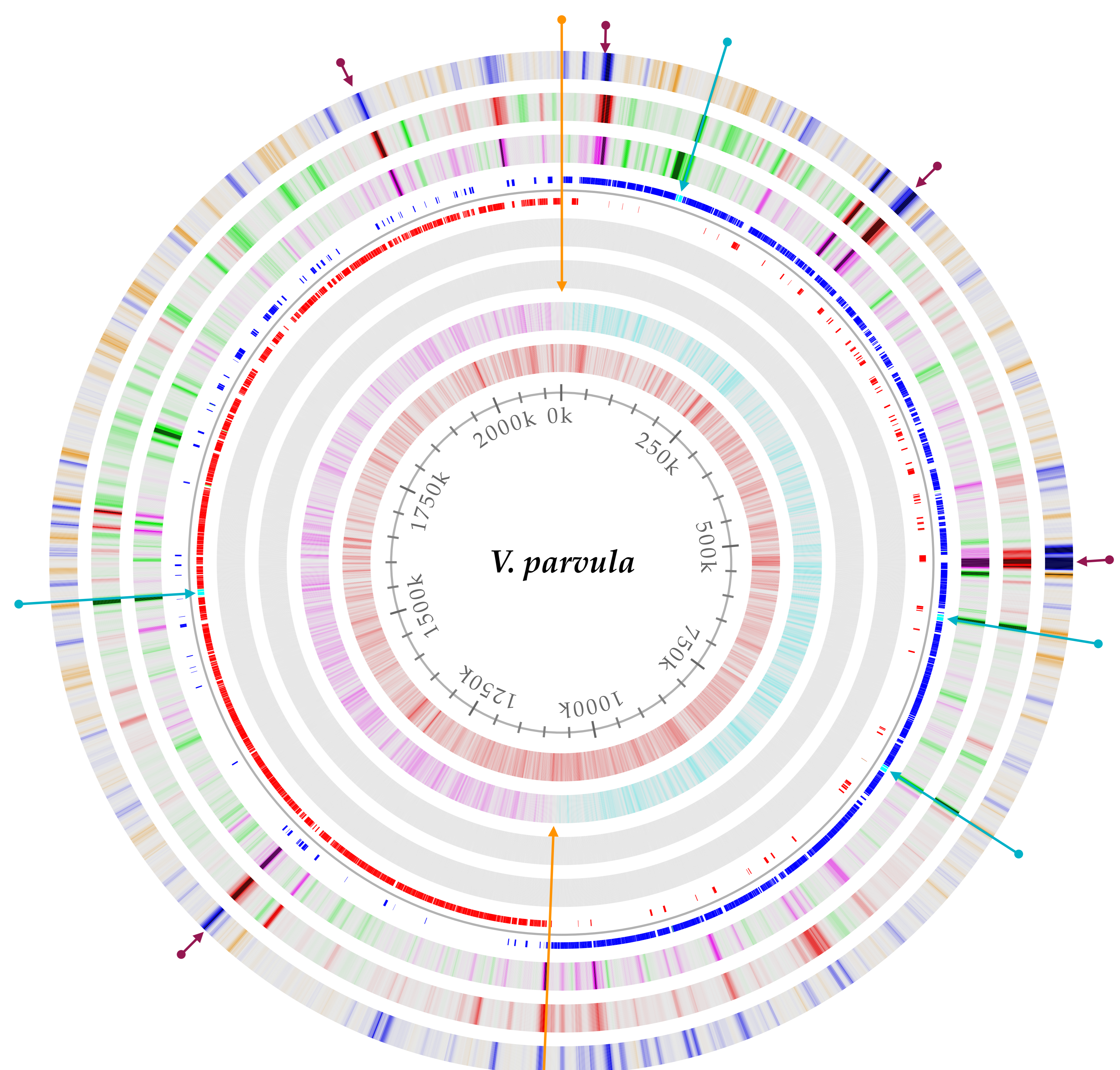

Supplement: Figure S1 — Genome atlases, DNA structures ( Figure 3 at High-Resolution). (PDF) [file pone.0060120.s001.pdf]

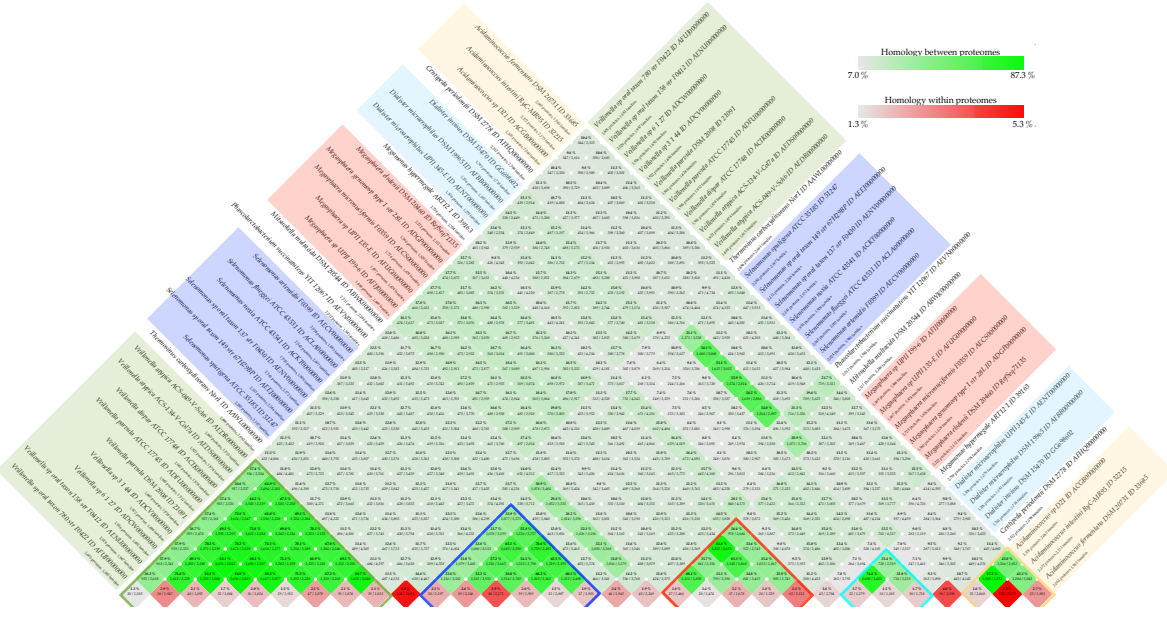

Supplement: Figure S2 — BLAST matrix ( Figure 6 at High-Resolution). (PDF) [file pone.0060120.s002.pdf]
